# Upper torso and pelvis linear velocity during the downswing of elite golfers

**DOI:** 10.1186/1475-925X-12-13

**Published:** 2013-02-11

**Authors:** Seung-Hui Beak, Ahnryul Choi, Seung-Wook Choi, Seung Eel Oh, Joung Hwan Mun, Heegoo Yang, Taeyong Sim, Hae-Ryong Song

**Affiliations:** 1 Raredisease Research Institute, Guro Hospital, Korea University, 97 Guro-Gil, Guro, Seoul 152-703, Republic of Korea; 2 Department of Bio-Mechatronic Engineering, College of Biotechnology and Bioengineering, Sungkyunkwan University, 300 Chunchun, Jangan, Suwon, Gyeonggi 440-746, Republic of Korea; 3 Department of Sports and Leisure, College of Human Ecology, Sungshin Women’s University, 249-1 Donseon 3-ga, Seongbuk, Seoul 136-742, Republic of Korea; 4 College of Pharmacy, The Catholic University, Bucheon, Gyeonggi, 420-743, Republic of Korea

**Keywords:** Golf, Linear velocity, Coupling, Cross-correlation, Downswing

## Abstract

**Background:**

During a golf swing, analysis of the movement in upper torso and pelvis is a key step to determine a motion control strategy for accurate and consistent shots. However, a majority of previous studies that have evaluated this movement limited their analysis only to the rotational movement of segments, and translational motions were not examined. Therefore, in this study, correlations between translational motions in the 3 axes, which occur between the upper torso and pelvis, were also examined.

**Methods:**

The experiments were carried out with 14 male pro-golfers (age: 29 ± 8 years, career: 8.2 ± 4.8years) who registered in the Korea Professional Golf Association (KPGA). Six infrared cameras (VICON; Oxford Metrics, Oxford, UK) and SB-Clinc software (SWINGBANK Ltd, Korea) were used to collect optical marker trajectories. The center of mass (CoM) of each segment was calculated based on kinematic principal. In addition, peak value of CoM velocity and the time that each peak occurred in each segment during downswing was calculated. Also, using cross-correlation analysis, the degree of coupling and time lags of peak values occurred between and within segments (pelvis and upper torso) were investigated.

**Results:**

As a result, a high coupling strength between upper torso and pelvis with an average correlation coefficient = 0.86 was observed, and the coupling between segments was higher than that within segments (correlation coefficient = 0.81 and 0.77, respectively).

**Conclusions:**

Such a high coupling at the upper torso and pelvis can be used to reduce the degree of motion control in the central nervous system and maintain consistent patterns in the movement. The result of this study provides important information for the development of optimal golf swing movement control strategies in the future.

## Background

Golf is a competitive sport where balls are hit into a series of holes on a golf course; thus, accuracy of direction and high distance of each swing is essential [1]. The goal of the game is to get the ball into a hole using the fewest number of strokes [2], which is possible with a high driving distance. The distance is determined by the initial ball speed at club impact, and the speed of the ball is a determined by the club head speed immediately before impact [1]. Therefore, many studies have attempted to develop strategies to increase the speed of the club head [3-5]. Most of these studies have attempted to analyze the mechanism of successful shots based on kinematic analysis of the upper torso and pelvis during golf swings [6-9].

According to Okuda et al. a successful golf swing is possible with the sequential movement of each human body segment, and it begins from the rotation in pelvis [10]. Bunn et al. suggested that the theoretically peak speed appears at the proximal segment first and the appearance of peak speed becomes delayed toward the distal segments, and sequential maintenance is a key to an efficient and successful swing [11]. This mechanism is for an efficient momentum transition from the proximal to the distal, and therefore, they concluded that the downswing of expert golfers start from the initial movement of the pelvis and upper torso. Several other experimental studies on sequential movements have also been reported [12-14], and the pattern behavior of expert golfers was found to be quite remarkable. Thus, the pelvis and upper torso are as significant as the segments in regards to maximizing the club head speed.

More recently, researchers have attempted to analyze and develop motion control strategies based on coordination between the pelvis and upper torso. According to the result of Kottke et al., when the human body tries a new behavior, it tends to use muscles inefficiently, which is controlled by the central nervous system [15]. However, by practicing the movements, the central nervous system changes muscle activation, and will develop an optimized strategy to perform certain motions. This process allows the body to become skillful on a specific motion [16]. In a highly redundant musculoskeletal system, most human body motions appear through combinations of many muscles, the central nervous system attempts to reduce the dimension of the control by activating functionally coordinated muscle groups rather than individual muscles. Therefore, establishing a simplified optimal control strategy through practice is possible [17]. If the strategy is expanded even slightly, simple control strategies appear as similar motor outputs by each axial movement in between and within segment [18]. A lot of researches have conducted related to the control strategy to improve the performance in golf swing [18-22].

According to Horan et al., a similar form of rotational motion was observed during the downswings of professional golfers, and a very similar relationship of peak and phase at the medial-lateral tilt and axial rotation velocity in the thorax was observed [19]. According to a recent study by the same authors, similarities in angular velocity, which is generated from each axis between and within segments in the head, thorax and pelvis were observed, and the angular velocity of each axis of the thorax and pelvis were highly correlated with an average r = 0.92 [18]. However, these recent studies only proposed control strategies based on the rotational angular velocity of the pelvis and thorax, and no study has performed a coupling analysis on the translation movement in between and within segments. Most human body movements are general motions that involve a combination of rotation and translation [23], and the golf swing also includes complex movements with simultaneous rotation and translation of each segment in three dimensional space. During the golf downswing, the only consideration of the rotational movements without the translational movements is incomplete control strategies. Thus, there is a need to develop a new motion control strategy that also includes translation.

Therefore, as a first study to develop a complex motor control strategies considering rotational and translational movement at once during golf downswing, the goal of this study was to analyze the coupling between translational motions that occur in 3 axis directions between and within two segments: upper torso and pelvis, during a skillful golfer’s downswing phase. We hypothesized that the linear speed and velocity in each direction of the upper torso and pelvis would be highly coupled.

## Methods

### Subjects & apparatus

The subjects used in this study included 14 professional golfers with no past history of musculoskeletal disorders. Each subject was a professional athlete (average career 8.2 ± 4.8 years) who registered at the KPGA (Korea Professional Golf Association), and all were right-handed golfers. Table 1 shows the body and swing characteristics of the professional golfers who participated in the experiment.

**Table 1:** Subject characteristics (S.D.)

**Gender**	**Age (years)**	**Height (m)**	**Weight (kg)**	**Handicap (strokes)**	**Peak clubhead speed (m/s**^**2**^**)**	**Downswing duration (sec)**
Males (n=14)	29 (8)	1.76 (7.9)	74.6 (9.3)	0 (0)	45.4 (3.9)	0.31 (0.04)

Six infrared cameras from VICON Inc. (VICON, Oxford Metrics, Oxford, UK) and SB-Clinic software (SWINGBANK Ltd, Korea) were used and the capture rate was set at 120Hz each recording. In order to remove the high frequency noise on acquired marker trajectories, a zero leg 4th low-pass Butterworth filter was applied, and the cutoff frequency of each marker was set from 6Hz to 10Hz through residual analysis [24].

### Procedures

**Figure 1:**
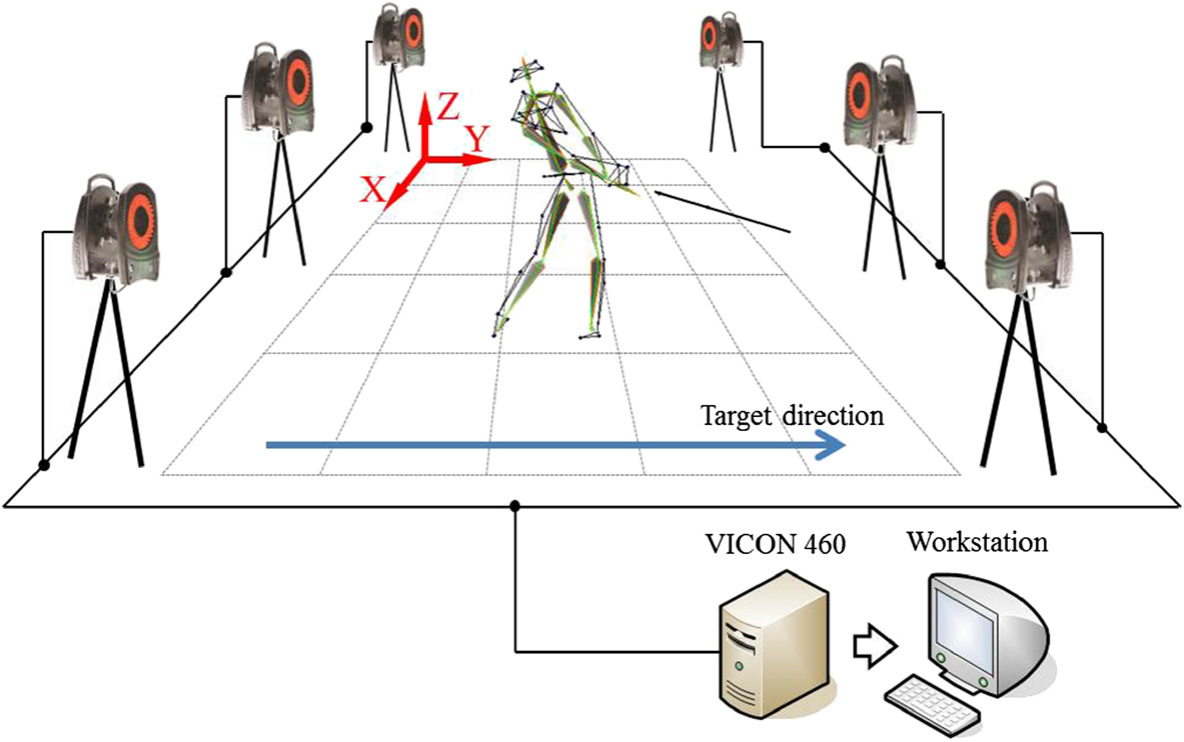
Golf swing analysis system.

For each subject, a total of nine markers were attached: 4 in the pelvis, 4 in the trunk and 1 on the club head. In the pelvis, markers were attached to the right and left, and anterior/posterior of superior iliac spine. For the trunk, markers were attached to the suprasternal notch and xiphoid process on the frontal, and C7 and T10 spinous process on the posterior. The marker points were part of the modified Helen Hayes marker set and the anatomical landmark between the trunk and pelvis. Before the actual experiment, each subject was allowed to warm up, which included large dynamic movement and static stretch exercise [25]. In addition, practice swings were performed to allow each subject to adapt to the laboratory environment. 5 swing repetitions per subject were performed, and 3 trials among the total were chosen randomly for data analysis. The swing analysis was limited to the downswing, which defined as an interval from the top of the backswing to the ball contact. The top of the backswing represents a transition where the pelvis stops to rotate clockwise and begins to rotate in the target direction [26], and the ball contact means a moment that the club head touches the ball. Data extracted on the downswing were normalized to 100% and analysis was performed. Figure 1 shows the overall golf swing analysis system used in this study.

### Upper torso and pelvis kinematics

**Figure 2:**
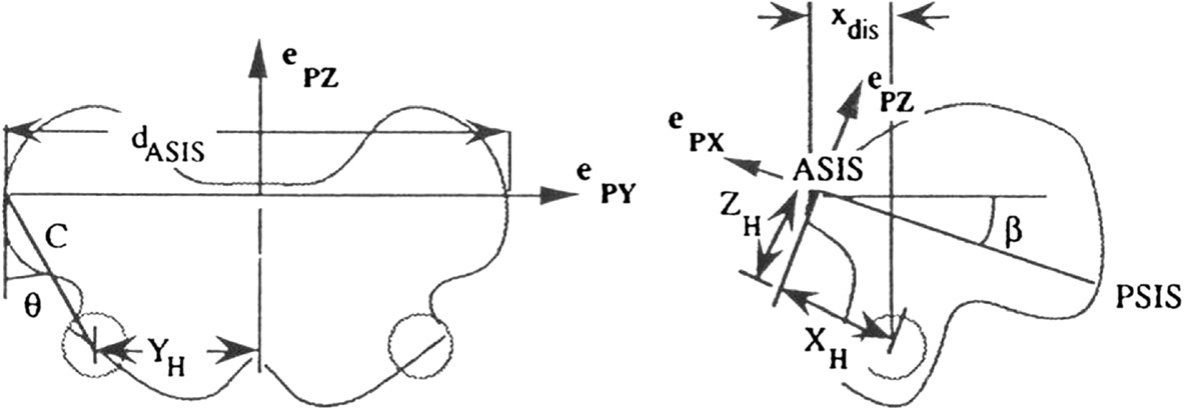
Hip joint centering geometry (scanned picture from Davis et al., 1991).

In order to calculate the translational speed of the trunk and pelvis, the center of mass (CoM) trajectories of each segment was extracted. The position of the CoM in each segment were calculated from the spatial location of the neck (C7/T1) joint, waist (L5/S1) joint and the hip joint on the leg. In case of the hip joint, a model based on a radiographic examination experiment by Davis et al. was used [27]. Based on the anatomical coordinate of the pelvis, the position of the hip joint was calculated as follows (Figure 2).

(1) $$X_{H}=\left[-x_{\text{dis}}-r_{\text{mar}\text{ker}}\right]\cos\beta +C\cos\theta \sin\beta$$

(2) $$Y_{H}=S\left[C\sin\theta -\frac{d_{\text{ASIS}}}{2}\right]$$

(3) $$Z_{H}=\left[-x_{\text{dis}}-r_{\text{mar}\text{ker}}\right]\sin\beta +C\cos\theta \cos\beta$$

Where, the coefficient values and definitions are as follow.

$$C=0.115\times \text{Leg}\;\text{legnth}\left(\mathrm{in}\;\text{meters}\right)-0.0153$$

θ=28.4°

β=18°

d_ASIS_: ASIS-to-ASIS distance (in meters), measured during the clinical examination

x_dis_: Anterior / posterior component of the ASIS/hip center distance (in meters) in the sagittal plane of the pelvis and measured during the clinical examination

r_marker_: Marker radius (in meters)

S:+1 for the right side, and −1 for the left side

For the neck (C7/T1) joint and waist joint (L5/S1), previously published equations were used [28]. In this previous work, the waist (L5/S1) joint was positioned at 9.04cm inside from the surface of the 5th lumber vertebra, and was tilted 6 degrees from horizontal with + clockwise. In addition, the neck (C7/T1) joint was positioned 7.47cm inside of the 7th thoracic vertebra, and tilted 25 degrees from the horizontal with + clockwise. Based on these previous studies, the positions of the hip, neck and waist joint can be calculated using a segment anatomical coordinate system.

These joint centers based on the anatomical coordinate system should be converted to the trajectories in the global coordinate system, and anatomical coordinate system of the pelvis and upper torso must be built previously. The anatomical coordinate system was determined based on the coordinates of the markers attached to the anatomical landmarks using the following formula [29].

(4) $${\vec{v}}_{1}={\vec{P}}_{\text{LASI}}-{\vec{P}}_{\text{RASI}}$$

(5) $${\vec{v}}_{2}={\vec{P}}_{\text{PSI}}-{\vec{P}}_{\text{RASI}}$$

Where, ${\vec{P}}_{\text{PSI}}$ represents the mid-point of ${\vec{P}}_{\text{LPSI}}$ and ${\vec{P}}_{\text{RPSI}}$

(6) $$\begin{array}{l} {\hat{g}}_{\text{pelvis}}=\frac{{\vec{v}}_{1}}{\left\|{\vec{v}}_{1}\right\|}\;{\hat{h}}_{\text{pelvis}}=\frac{{\vec{v}}_{1}\times {\vec{v}}_{2}}{\left\|{\vec{v}}_{1}\times {\vec{v}}_{2}\right\|}\;{\hat{f}}_{\text{pelvis}}={\hat{g}}_{\text{pelvis}}\times {\hat{h}}_{\text{pelvis}} \end{array}$$

(7) $$\left[T_{\text{pelvis}}\right]=\left[\begin{array}{ccc} a_{11} & a_{12} & a_{13}\\ a_{21} & a_{22} & a_{23}\\ a_{31} & a_{32} & a_{33} \end{array}\right]=\left[\begin{array}{ccc} {\hat{f}}_{\text{pelvis}} & {\hat{g}}_{\text{pelvis}} & {\hat{h}}_{\text{pelvis}} \end{array}\right]$$

**Figure 3:**
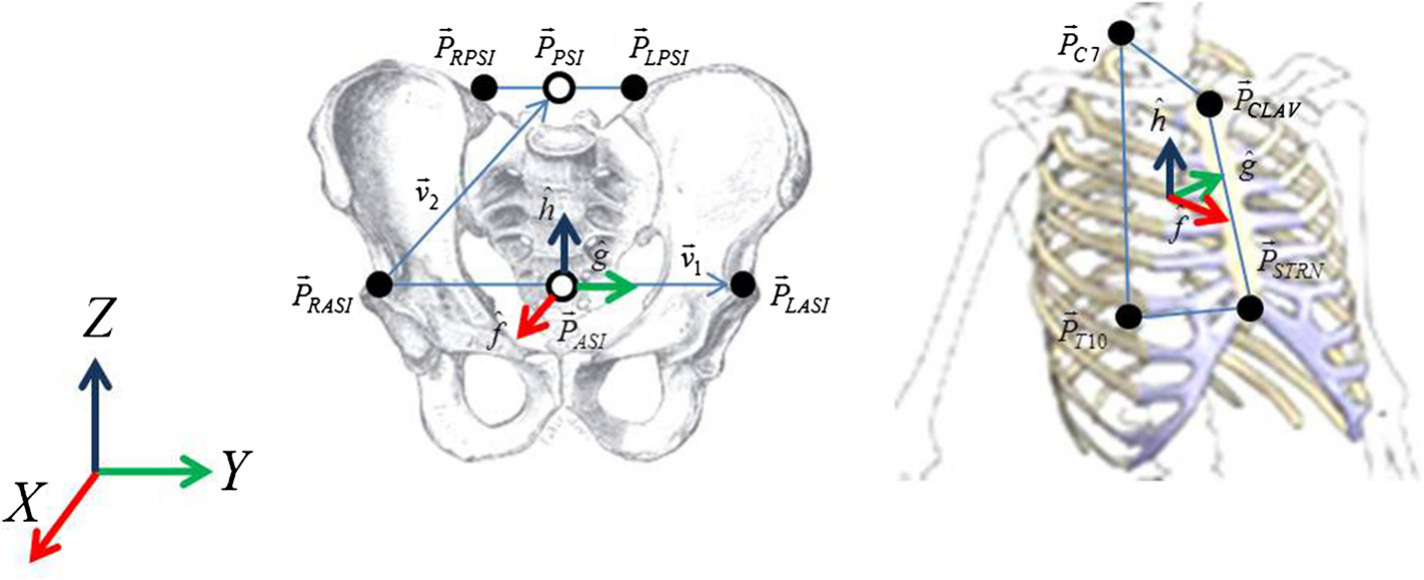
Anatomical coordinate system for the pelvis and upper torso.

Here, [*T*_*pelvis*_] represents the transformation matrix between the global and anatomical coordinate system of the pelvis. In the case of the upper torso and pelvis, a transformation matrix was generated from the trajectories of markers attached to the anatomical landmarks, and the anterior/posterior direction was set as the x-axis, medial/lateral direction was set as the y-axis and the up/down direction was set as the z-axis. Figure 3 shows the anatomical coordinate system.

The calculated anatomical coordinate transform matrix was used to recover the trajectories of the joint center during the golf swing based on the global coordinate system.

(8) P→GLASI=TpelvisLGP→LLASI+P→GASI

The CoM of the each segment was used to construct a line that connected the joint center and were calculated as described by Winter [30]. In addition, the velocity at each direction was calculated by applying the forward difference method, and the linear speed was calculated by applying the square root of the squared sum of the linear velocities in each direction.

### Data analysis

In this study, the peak values of linear speed in the pelvis and trunk, and the linear velocity in the 3-dimensional axis were calculated. Generally, speed can be used as a scalar value and can measure the change in the segment motion occurs with time; however, this value cannot be used to describe the components in each direction. The velocity, as vector components, can describe information about each direction of body segments. Therefore, linear speed and velocity was used for the analysis.

Typically, cross-correlation is similar in nature to the convolution operation of two functions, and can analyze the similarity of two signal patterns under continuous time [31]. It can be calculated by shifting one of signal series relative to the other. The number of data points that the series is shifted is represented the time-lag. The equation generally used is as follows:

(9) $$r\left(l\right)=\frac{\sum_{i}\left[\left(x_{i}-\bar{x}\right)\left(y_{i-l}-\bar{y}\right)\right]}{\sqrt{\sum_{i}{\left(x_{i}-\bar{x}\right)}^{2}}\sqrt{\sum_{i}{\left(y_{i-l}-\bar{y}\right)}^{2}}}$$

Where, the time-lag is denoted by l and r represents the correlation coefficient. Total 199 (2*100-1) correlation coefficients were calculated within two signal series normalized into 100, and the ratio to have positive correlation coefficients among the maximum absolute correlation coefficients of the two signal series in overall time was obtained. In addition, time-lag (the number of data points), when maximum absolute correlation coefficient appears, was calculated. Coupling in linear speed and velocity at intersegments was analyzed based on the correlation coefficient of each direction in the pelvis and upper torso and coupling in each segment was determined based on the linear velocity of the 3 axis directions correlation coefficient. A correlation coefficient higher than 0.8 indicates that the two patterns have high coupling strength, whereas a value between 0.7 and 0.8 and below 0.7 indicate the patterns have a moderate and low coupling, respectively [32]. These data analyses were performed using MATLAB version 6.5.0 (The MathWorks, Natick, MA, USA). Also, differences in peak velocity with time, and differences in peak velocity value at each segment and axis was analyzed by ANOVA (Tukey's HSD) and the t-test. The significant level was set at p < 0.05.

## Results

**Figure 4:**
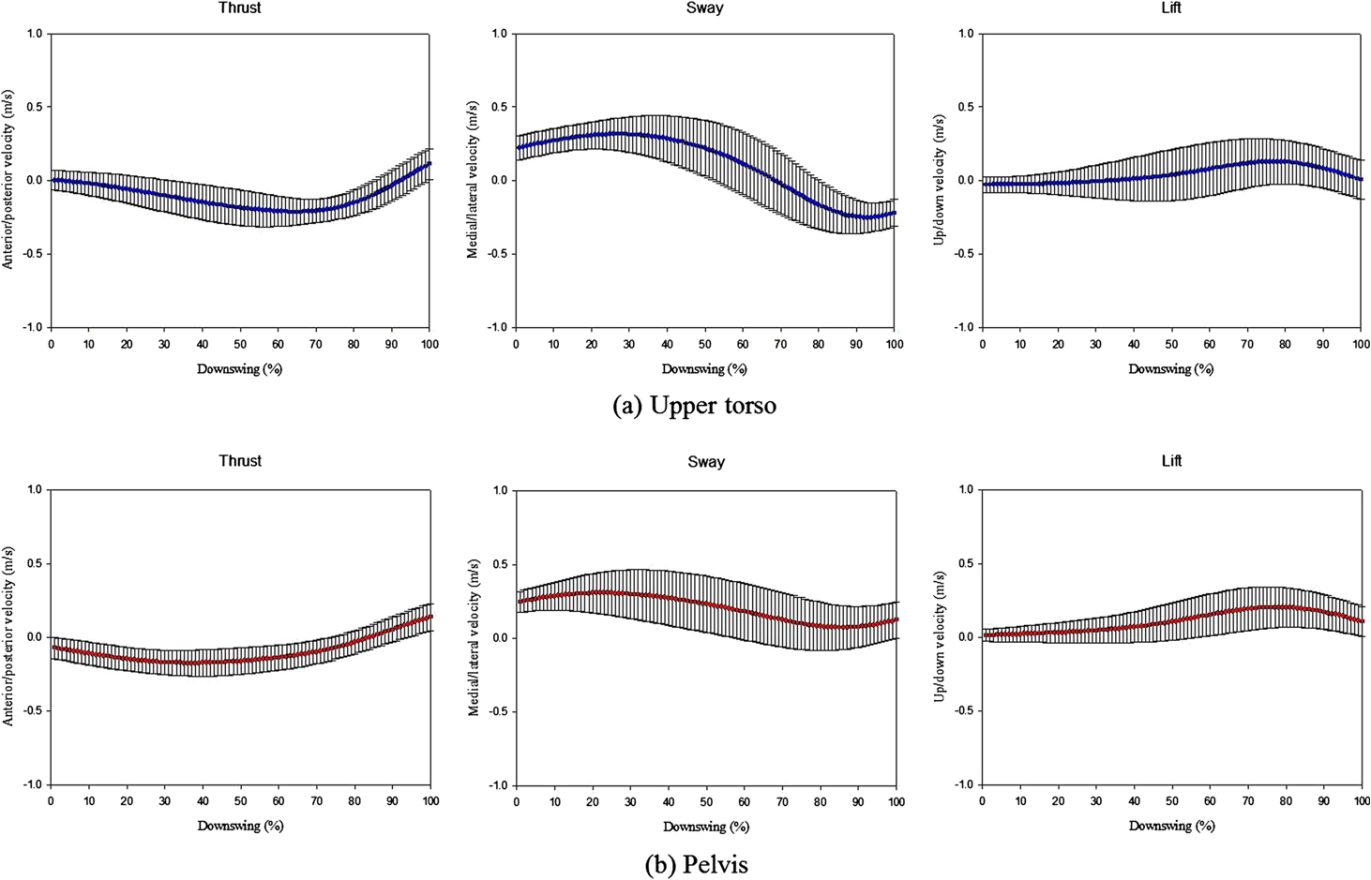
Ensemble average for the pelvis and upper torso linear velocity during a golf downswing (14 pro-golfers).

Figure 4 shows the results of the linear velocity in the 3-axis at the pelvis and upper torso during the downswing of 14 pro-golfers. The patterns in each direction were similar at the pelvis and upper torso, but the time and the value where the peak points occurred were different. In addition, a distinctive pattern and the highest range of velocity were observed in the medial/lateral direction for the upper torso and pelvis, respectively.

Table 2 shows the peak value for the linear speed and velocity, and the time it occurred in the pelvis and trunk during the downswing of 14 pro-golfers. Also, when looking at the linear velocity in each direction, the minimum medial/lateral linear velocity was higher at the pelvis rather than the upper torso, and a higher maximum and minimum vertical linear velocity was found at the pelvis (p < 0.001). As for the anterior/posterior direction, the time when the minimum peak value occurred was earlier at the pelvis than the upper torso (p < 0.001), and the same results were observed in the medial/lateral (p < 0.035) and up/down (p < 0.045) directions. Within each segment, the maximum medial/lateral linear velocity was higher than that in the anterior/posterior and up/down directions, and the minimum vertical linear velocity was higher than that in the anterior/posterior and medial/lateral directions (p < 0.001). Also, the maximum linear velocity of the pelvis and upper torso occurred in sequence: medial/lateral, up/down, anterior/posterior direction, and statistically significant differences were observed (p < 0.001). In the case of the time for the minimum peak at the pelvis and upper torso, both occurred in a sequence from up/down, anterior/posterior, medial/lateral, and a statistically significant difference was only observed in the medial/lateral direction (p < 0.001).

**Table 2:** Amplitude of maximum and minimum speed and velocity for each segment and direction with respect to the timing of peaks during the downswing (S.D.)

	**Max/min speed (m/s)**	**Downswing (%)**	**Direction**	**Max/min velocity (m/s)**	**Downswing (%)**
**Upper torso**	0.440 (0.11)	59 (26)	Anterior/posterior	0.125 (0.09)^b^	94 (21)^b,c^
				−0.245 (0.09)^c^	66 (11)^**,b^
			Medial/lateral	0.345 (0.13)	26 (12)^a,c^
	0.203 (0.08)	49 (45)		−0.267 (0.11)^**,c^	93 (5)^*^
			Up/down	0.169 (0.13)^**,b^	71 (24)^a,b^
				−0.108 (0.09)^**^	48 (44)^*,b^
**Pelvis**	0.434 (0.11)	47 (23)	Anterior/posterior	0.136 (0.09)^b^	96 (16)^b,c^
				−2.030 (0.08)	39 (16)^b^
			Medial/lateral	0.343 (0.13)	22 (16)^a,c^
	0.189 (0.06)	65 (41)		0.041 (0.14)^a^	76 (27)
			Up/down	0.231 (0.11)^b^	78 (12)^a,b^
				−0.022 (0.07)^a^	28 (36)^b^

* and ** denote *p<0.05 *and *p<0.01 *respectively between upper torso and pelvis.

^a^significantly different from anterior/posterior, ^b^significantly different from medial/lateral, ^c^significantly different from up/down direction.

Table 3 shows the results of the coupling and the phase difference in linear speeds and velocities between the upper torso and pelvis of each golfer during the downswing. A strong coupling strength was observed for the upper torso-pelvis linear speed, and the lowest coupling (r = 0.75) was found in the medial/lateral direction. Also, the ratio of having a positive r in linear velocity was 100%; thus, the coupling occurred in the positive direction for all swings and the lowest positive coupling was found in the medial/lateral direction (79%). Overall, the linear velocity and speed in each direction of the upper torso and pelvis had a positive coupling. In the case of the phase difference, the pelvis had the highest precedence (81%) on average relative to the other directions, except for the up/down direction.

**Table 3:** Maximum cross-correlation coefficients and phasing for between segment analyses (S.D.)

**Upper torso-pelvis**	**Max. correlation coefficient**	**Positive correlation coefficient (%)**	**Phasing**	**- : 0 : + (ratio)**
Anterior/posterior	0.84 (0.15)	86	26 (18)	3 : 0 : 39 (93%)
Medial/lateral	0.75 (0.16)	79	3 (13)	9 : 0 : 33 (79%)
Up/down	0.88 (0.11)	86	−7 (23)	21 : 9 : 12 (50%)
Speed	0.97 (0.02)	100	11 (18)	9 : 3 : 30 (71%)

Max. correlation coefficient: The maximum absolute value among the correlation coefficients of two types of data series from the overall time domain.

Positive correlation coefficient (%): The ratio to have positive correlation coefficients among the maximum absolute correlation coefficients of the two types of data series in the overall time domain.

Phasing: The time-lag (the number of data points) when maximum absolute correlation coefficient appears.

- : 0 : + (ratio): The number and ratio of negative (upper torso is leading), zero (occur simultaneously) and positive (upper torso is lagging) phasing value among the total trials (14 subjects x 3 trials).

Table 4 shows the coupling and phase difference in linear velocities within each segment in the upper torso and pelvis, respectively. The average r was 0.79, which indicates that there was a lower coupling within segments, and better coupling for the pelvis than upper torso. When coupling in directions are considered, the upper torso and pelvis both have higher value in the anterior/posterior and up/down directions with r values of 0.79 and 084, respectively. Also, the ratio of having a positive r was much higher for the pelvis than the upper torso. The anterior/posterior and medial/lateral direction in the pelvis had a 7% ratio of having a positive r and the correlation was in the negative direction, and the medial/lateral and up/down direction had the highest positive ratio (86%). Similar results were observed between the upper torso and pelvis in regards to the phase difference, where the medial/lateral peak value of upper torso was the highest (38%) relative to the frontal/posterior direction throughout the swings (100%). The medial/lateral (56%) was higher than the up/down directions, and the peak value leading patterns were observed throughout the whole swings (100%), as was observed for the upper torso.

**Table 4:** Maximum cross-correlation coefficients and phasing for within segment analyses (S.D.)

		**Max. correlation coefficient**	**Positive correlation coefficient (%)**	**Phasing**	**- : 0 : + (ratio)**
Upper torso	A/P – M/L	0.75 (0.15)	14	38 (11)	0 : 0 : 42 (100%)
	A/P – U/D	0.79 (0.19)	21	−6 (24)	33 : 3 : 6 (79%)
	M/L – U/D	0.77 (0.15)	50	−45 (27)	21 : 9 : 12 (50%)
Pelvis	A/P – M/L	0.79 (0.15)	7	16 (12)	3 : 0 : 39 (93%)
	A/P – U/D	0.84 (0.08)	21	−39 (21)	36 : 0 : 6 (86%)
	M/L – U/D	0.80 (0.08)	86	−56 (20)	42 : 0 : 0 (100%)

A/P: Anterior/posterior, M/L: Medial/lateral, U/D: Up/down direction.

## Discussion

Pelvis and upper torso, as segments that initiate the movement during a golf downswing, will move in an appropriate magnitude and sequence to maximize the club head speed. In order to better understand the role of the pelvis and upper torso in motion control, we analyzed the correlation between the linear speed and velocity of the pelvis and upper torso during the downswings of pro-golfers. To determine this, the peak values of the linear speed and velocity, and the time when these peaks occurred were extracted. A similar trend was observed in each direction of the pelvis and upper torso. In addition, the 3-axis direction of each segment was assessed using cross-correlation analysis. Previous studies only examined the rotational motions in each segment and similarity analysis for the translational motions has yet to be performed. Thus, the results obtained in this study will provide basic information to better understand the motion control strategy during the downswing of professional golfers.

The maximum and minimum speed in the upper torso was larger than in the pelvis, but this was not statistically significant. However, differences were observed in the axis linear velocity between the medial/lateral and up/down directions. In particular, we examined the velocity range (maximum value - minimum value) in the medial/lateral direction (0.612 ± 0.10 m/s) of the 14 pro golfers and the maximum was value differed by 3.335 m/s when compared with the other axis ranges. These differences are believed to be the mechanism by which maximum power is delivered to the ball at impact, and thereby a weight shift process naturally occurs. According to Burden et al., the swing speed can be increased when the human CoM moves into the ball direction at impact [13], and Okuda et al. concluded that there was a significant weight shift transition phenomena during the downswing for professional golfers [10]. The trunk, in fact, contains most of the mass among the segments that comprise the body [30], and the weight transition of the pro-golfers is considered to be close related with shift in the torso CoM and variation of velocity; therefore, intensive studies will be needed to clarify these relationships.

The similarity analysis between the pelvis and upper torso resulted in an average r value of 0.86, and the highest value was observed for the linear speed (r = 0.97 ± 0.02). This likely occurred because the patterns of the pelvis and trunk were similar to each other during the overall downswing phase. However, the correlation coefficients in the medial/lateral direction between upper torso and pelvis had the lowest value (r = 0.75 ± 0.16) compared to the anterior/posterior and up/down direction. This means that the motion control strategy that occurs in the pro-golfers are not simple compared to the other axis. According to the result of Horan et al., the pelvis and thorax exhibited the most movement in the medial/lateral direction during the downswing [19]. Therefore, the medial/lateral direction showed a lot more motion when compared to the other axis.

The difference in the timing of the peak value was observed in the anterior/posterior direction, where the pelvis lead the upper torso (93% rate of the pelvis movement precedence to the upper torso in entire trials of 14 professional golfers), and leading and lagging appeared diversely in the up/down direction. Also, the phase differences within the segment were as follows: in the anterior/posterior-medial/lateral directions, the medial/lateral direction led with an average of 97% at the pelvis and upper torso. In a previous studies [1,12], the pelvis was reported to have led the upper torso in rotational angular velocity on the axial axis, and such a sequential movement was proposed as a mechanism for optimizing the speed of the club head. Nevertheless, these previous studies have only focused on the rotational motion, and no study has examined the translational motion of each segment. Therefore, based on the phase difference result of this study, the sequence of movement during the downswing can be calculated, and the appropriate motion control strategy can be developed in the future.

Final goal of control strategy in the golf is to improve the swing performance. Therefore, the purpose of studies related to the motor control during golf swing is to find variables that exhibited consistency (absolute invariance) and variability in skilled golfers, and use the consistent variables as a golf teaching tool. Bradshaw et al. proposed that absolute invariance seem to be more effective technique in the main swing phases such as the top of the backswing [21]. Similarly, coupling between the upper torso and pelvis was higher at pro-golfers, and these consistencies can be used as useful information during a golf teaching. Variables with high variability in the pro-golfers, however, variety of movement strategies can be existed and those are caused by variety of reasons, such as personal characteristics or individual differences [22]. Therefore, higher order control strategies including individual differences are considered to be required for the stabilization on golf swing.

This study was performed with an aim that establishing a proper control strategy of the pelvis and torso in golf downswing, and analyzed the control strategies of the linear movement by the coupling between the linear velocity and speed in the skilled golfers’ upper torso and pelvis. In the future, simplified functionality controlled by the central nerve system for a proper downswing movement can be analyzed with coupling analysis between the translational and rotational movements, and the results can be used to establish a complex control strategy of the pelvis and torso motions. In addition, complex sequence can be established by the phasing differences between each linear velocity along with those of the rotational movements, which proposed in the published results [1,12].

The results of this study can be summarized as follows.

1. During the downswing of pro-golfers, the pelvis and upper torso translational movement showed a high coupling strength between the two segments with an average r=0.86. This strategy likely reduces and simplifies the dimension of motion control in the central nervous system, and may be used to maintain a consistent motion pattern.

2. The coupling within a segment in the 3-axis directions of the pelvis and upper torso had an average r=0.79, which is low when compared to the coupling between segments. The low coupling between pro-golfers weakens the possibility of a specified coordinate pattern, and thus there are probably many different motion control strategies. Therefore, since there can be various coordination patterns within each pelvis and upper torso segment, there can be flexibility in the future training of amateur golfers.

3. In regards to the linear velocity, phase differences in the pelvis and upper torso were as follows; the pelvis led the upper torso in the frontal/posterior direction, and medial/lateral direction led the frontal/posterior direction. The phase differences can be used to calculate the sequence of the translational motion during the downswing, and also can be used to help establish an optimal motion control strategy along with rotational motion.

Golf swings are an exercise to maximize the speed of the club head while releasing the accumulated energy oriented in rotational motion of each segment [8]. The rotational motion in the pelvis and upper torso showed a very high coupling strength with an average r=0.92 in the study by Horan et al. [18]. However, to establish a specific motion control strategy, both the rotational and transitional motions must be considered. In this study, the coupling of transitional motions at the pelvis and upper torso were analyzed during the downswing, and the results of this study will be important for the development of an optimal exercise control strategy. However, this study has limitations in regards to lack of analysis of unskilled players. Analyzing the swing control strategy of amateur golfers relative to professional players is needed, and studies to establish an optimum swing control strategy by correlation and phase analysis of rotational and translational motion is necessary.

## Competing interests

All authors declare that they have no competing interests.

## Authors’ contributions

S-HB and AC made substantial contributions to analysis of data and performed the statistical analysis. Also, they were involved in drafting the manuscript. SEO, TS and HY conducted an overall experiments and data acquisition. Prof. S-WC made contribution to conception of this study. Prof. JHM participated in the design of the study. Prof. H-RS made substantial contributions to interpretation of data. In addition, Prof. S-WC, JHM and HRS made revising manuscript critically and gave the final approval of the manuscript. All authors read and approved the final manuscript.
